# Relationship Between Blood Urea Nitrogen to Albumin Ratio and Risks of Mortality and Delirium in Pancreatitis Patients: A Retrospective Analysis of the MIMIC‐IV Database

**DOI:** 10.1155/grp/5512602

**Published:** 2026-10-15

**Authors:** Shuning Duan, Jie Xu, Yiwen Zu, Peicong You, Qiuyi Wang

**Affiliations:** ^1^ Department of Critical Care Medicine Tianjin Hospital Tianjin China tjorthop.org

**Keywords:** blood urea nitrogen to albumin ratio, delirium, mortality risk, pancreatitis, prognostic prediction

## Abstract

**Background and Objective:**

This study is aimed at evaluating the predictive value of the blood urea nitrogen‐to‐albumin ratio (BAR) for both mortality and delirium risk in patients with pancreatitis. Pancreatitis carries a substantial mortality risk and significant long‐term sequelae, with delirium being a severe neurological complication, underscoring the clinical need for effective prognostic markers.

**Methods:**

A retrospective cohort study was performed using the MIMIC‐IV v3.0 database. A total of 1748 ICU patients meeting diagnostic criteria for pancreatitis (ICD‐9/10 codes: 577.0, 577.1, K85) with documented baseline blood urea nitrogen (BUN) and albumin levels were included. BAR was calculated (BUN[mg/dL]/Albumin[g/dL]) and patients were stratified into quartiles (Q1: BAR < 3.0; Q2: BAR 3.0–5.0; Q3: BAR 5.0–9.2; Q4: BAR ≥ 9.2). The primary endpoints were all‐cause mortality and delirium occurrence (identified using ICD‐9 codes 293.0, 293.1, and ICD‐10 codes F05, F05.9). Multivariable Cox proportional hazards regression (mortality) and logistic regression (delirium) were employed, adjusting for demographics, illness severity scores (GCS, SAPS II, OASIS, and Charlson Comorbidity Index), and comorbidities. Restricted cubic splines (RCS) were used to explore nonlinear associations.

**Results:**

Higher BAR levels significantly correlated with greater disease severity: compared with Q1, Q4 exhibited a higher Charlson Comorbidity Index and mortality rate. BAR was an independent predictor of delirium; each 1‐unit increase in BAR was associated with a 2.7% increased risk of delirium (adjusted OR = 1.027, 95% CI: 1.009–1.044), and patients in Q4 had a significantly higher risk compared with those in Q1 (adjusted OR = 1.882, 95% CI: 1.157–3.108). After multivariable adjustment, BAR was not significantly independently associated with mortality (Q4 adjusted HR = 1.022, *p* = 0.8). RCS analysis revealed BAR > 9.0 as a high‐risk threshold, characterized by delirium OR > 1.78 and mortality HR > 1.97. The BAR quartile model yielded AUCs of 0.635 and 0.599 for predicting mortality and delirium, respectively, indicating modest predictive ability.

**Conclusion:**

BAR independently predicts delirium risk and reflects disease severity in pancreatitis patients, but does not demonstrate significant independent predictive value for mortality after accounting for confounders. The identified threshold (BAR > 9.0) supports the utility of BAR as a supplementary biomarker for delirium risk stratification in this population.

## 1. Introduction

Pancreatitis is an inflammatory condition of the pancreas that often leads to systemic complications [1]. According to the GBD 2017 study, over 60 million prevalent cases of pancreatitis were reported globally in 2017, with an inhospital mortality of 1%–5% for acute pancreatitis (AP) and up to 20%–40% for severe cases [2]. Beyond its high mortality, pancreatitis incurs substantial healthcare costs and often leads to long‐term sequelae among survivors, including immune dysfunction, cognitive decline, psychiatric impairments, and reduced quality of life. Additionally, pancreatitis significantly contributes to the development of bacterial antimicrobial resistance. In the US, from 1997 to 2012, the hospital charge increased from $15,433 to $33,744 [3]. Total hospitalization costs per patient sometimes exceed $2.6 billion in aggregate annually [4]. A meta‐analysis showed that the mean age of onset is about 56 years and demonstrates a steadily rising incidence over time in most countries of the Western world [5]. Accurate prediction of mortality risk enables early intervention, improving prognosis and reducing the overall healthcare burden associated with this condition.

Delirium is a common and severe cerebral dysfunction in intensive care units (ICUs), characterized by impaired attention, altered consciousness, and deficits in cognitive functions such as memory, orientation, and language. It is associated with increased morbidity and mortality, prolonged hospital stays, higher healthcare costs, and persistent cognitive impairment after discharge [6, 7]. Neurological symptoms in pancreatitis, although less common, can include delirium and are associated with more severe disease and higher mortality [8]. One study showed that among 830 patients with AP, 151 (18.1%) developed at least one extrapancreatic complication (EPC). The prevalence of delirium was 14.5% in AP patients with EPC [9]. Studies report that delirium can affect a significant proportion of severely ill pancreatitis patients and that the symptom not only elevates mortality but also leads to long‐term cognitive and psychological impairments [10, 11]. Furthermore, it has been recognized that brain injury following pancreatitis is usually associated with a significantly increased risk of depression within postpancreatitis recovery. Although this finding was primarily derived from studies on chronic pancreatitis, it may also be applicable to patients recovering from AP, given the overlapping systemic inflammatory responses and neurological complications associated with both conditions. [12, 13].

BUN, an end product of protein metabolism excreted by the kidneys, is widely used clinically to assess renal function and metabolic status. BUN levels are frequently elevated in severe systemic inflammatory conditions because of impaired renal perfusion, inflammation‐induced renal injury, and metabolic disturbances [14]. Conversely, serum albumin (Alb) reflects nutritional status and inflammatory response. During pancreatitis, pro‐inflammatory cytokines inhibit Alb synthesis, whereas increased vascular permeability leads to Alb loss, resulting in hypoalbuminemia [15]. Blood urea nitrogen‐to‐albumin ratio (BAR), combining these two parameters, provides complementary physiological and pathological information and has been increasingly investigated as a prognostic marker in various diseases [16–18]. Two recent studies have identified BAR as an independent risk factor for mortality in septic ICU patients [19, 20]. However, the precise relationship between BAR and pancreatitis outcomes remains unclear, and the predictive value of BAR for pancreatitis‐related delirium is yet to be established. Therefore, this study performs a retrospective cohort analysis using the MIMIC‐IV database to evaluate the prognostic utility of BAR for mortality and delirium risk in pancreatitis patients during ICU admission.

## 2. Method

### 2.1. Database Selection

This research utilized a historical cohort approach, drawing on anonymized clinical data sourced from the MIMIC‐IV v3.0 database. As an evolution beyond MIMIC‐III, this version provides comprehensive ICU patient records from Beth Israel Deaconess Medical Center, covering the years 2008–2022. The database includes essential patient information, vital signs, diagnostic codes, laboratory measurements, treatment details, and survival data. The MIMIC‐IV database is accessible through the PhysioNet platform. To gain access to and utilize the data, one of the study′s authors, Shuning Duan, completed the Collaborative Institutional Training Initiative (CITI) courses for human research (Certificate Number:12845394).

### 2.2. Criterion for Patients

Patient selection followed these inclusion criteria: (1) Patients diagnosed with pancreatitis according to ICD‐9 and ICD‐10 codes; (2) Documented baseline levels of both BUN and Alb, enabling calculation of the BUN‐to‐Albumin (BAR); (3) Patients stayed in ICU and hospital > 24 h; (3) Patients first admitted in hospital and ICU; (4) 18 < patient age < 85. Datasets exhibiting less than 20% missing values in continuous variables underwent mean imputation using the study population′s averages to maintain analytical power. From this cohort of 1748 retrospectively enrolled patients, BAR was calculated as BAR = BUN/Alb. Subjects were subsequently stratified into tertiles based on RPR interquartile ranges (IQRs): Q1 (< 3.0), Q2 (3.0–5.0), Q3 (5.0–9.2), and Q4 (> 9.2) (Figure 1). Whether a patient has delirium is determined based on the CAM‐ICU score [21]. According to the CAM‐ICU scoring system, if a patient meets the combined criteria of Feature 1 and 2, plus 3, or Feature 1 and 2, plus 4, they will be classified into the delirium group. In the MIMIC‐IV database, diagnosis of pancreatitis was according to the International Classification of Diseases, Ninth Revision (ICD‐9) codes (beginning with 5770) or 10th revision (ICD‐10) codes (beginning with K850–K859).

**Figure 1:**
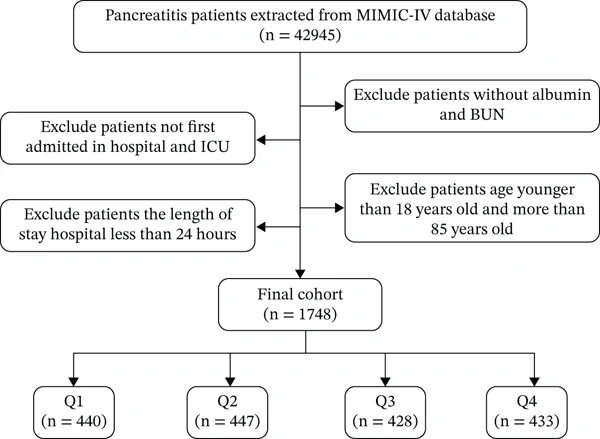
Work flow for patient selection.

In our study, we identified patients using all pancreatitis‐related ICD codes, including acute, chronic, and recurrent pancreatitis. AP was the predominant type, accounting for 1356 of 1748 enrolled patients (77.6%). Due to the limitations of ICD coding in distinguishing chronic from recurrent pancreatitis in retrospective analyses, detailed counts for these subtypes were not separately reported.

### 2.3. Clinical Outcomes

The primary endpoints of this study comprised all‐cause mortality and delirium.

### 2.4. Variables

The analysis incorporated the following variables:

1. Demographic data: patient age and gender;
2. Comorbid conditions: documented pre‐existing illnesses were categorized using standard classifications, including myocardial infarction, congestive heart failure, peripheral vascular disease, cerebrovascular disease, chronic pulmonary disease, rheumatic disease, peptic ulcer disease, mild liver disease, severe liver disease, diabetes (both with and without chronic complications), paraplegia, renal disease, and malignancies (solid tumors, hematologic malignancies, and metastatic solid tumor);
3. ICU severity metrics: Validated illness severity scores captured at ICU admission (Glasgow Coma Scale [GCS], Oxford Acute Severity of Illness Score [OASIS], Sequential Organ Failure Assessment [SOFA], Simplified Acute Physiology Score II [SAPS II], and Charlson Comorbidity Index);
4. Laboratory parameters: Measured indices upon or near ICU admission (International Normalized Ratio [INR], prothrombin time [PT], partial thromboplastin time [PTT], anion gap, bicarbonate, BUN, Alb, calcium, chloride, creatinine, glucose, sodium, potassium, hematocrit, hemoglobin, mean corpuscular hemoglobin [MCH], mean corpuscular hemoglobin concentration [MCHC], mean corpuscular volume [MCV], platelet count, red blood cell count [RBC], red cell distribution width [RDW], and white blood cell count [WBC]).

### 2.5. Statistical Method

Baseline characteristics of the study cohort were summarized according to the different outcome groups. Categorical variables are presented as frequency counts and percentages. Continuous variables are reported as either mean ± standard deviation or median with IQR, chosen based on data distribution. Group comparisons for continuous metrics employed either ANOVA or the Wilcoxon rank‐sum test, as appropriate. Differences in categorical characteristics across outcome groups were evaluated using the chi‐square test or Fisher′s exact test.

Survival probability estimates based on RPR‐defined groups were generated using Kaplan–Meier (K–M) curves. Incidence rates for the primary endpoint across these groups were derived from these curves, and statistical significance between groups was assessed with the log‐rank test.

Assessment of the independent association between the RAR index and the risk of all‐cause mortality was conducted using Cox regression analysis, and the risk of delirium was assessed using logistic regression analysis. The index was input as a categorical variable and a continuous variable. Four models were used in the regression analysis. Adjustments were made to the multivariable models, as follows: no adjustments were made to Model 1; adjustments were made for Model 2 according to age, gender; Model 3 was adjusted according to Model 2 by adding GCS, SAPS II, OASIS, SIRS, and Charlson Score, with multicollinearity assessed using variance inflation factor (VIF) (threshold < 5). A restricted cubic spline (RCS) regression model was then used to explore potential nonlinear relationships between BAR levels and outcome risks. Statistical significance for trends across the categorized BAR levels (Q1–Q4) was determined. To assess effect modification, stratified analyses and interaction testing (using likelihood ratio tests) were conducted for key covariates: gender, age (< 65 vs. ≥ 65 years), hospital length of stay (LOS), ICU LOS, heart failure, myocardial infarction, peripheral vascular disease, cerebrovascular disease, chronic pulmonary disease, mild liver disease, renal disease, and metastatic solid tumor.

## 3. Results

### 3.1. Baseline Information

A total of 1748 patients were included in this study. The baseline demographic and clinical characteristics of the enrolled patients are presented in Table 1. Among them, 193 patients had delirium. According to the quartiles of the BAR, they were divided into four groups: Q1 (< 3.0, *n* = 440), Q2 (3.0–5.0, *n* = 447), Q3 (5.0–9.2, *n* = 428), and Q4 (> 9.2, *n* = 433). There were significant differences in demographic characteristics, laboratory indicators, and clinical outcomes among the four groups of patients (*p* < 0.05). Patients in the higher BAR group were significantly older (median 53.9 years in Q1 vs. 65.8 years in Q4, *p* < 0.001), had a higher proportion of males (52.0% in Q1 vs. 61.0% in Q4, *p* = 0.024), and had a significantly longer hospital stay (8.8 days in Q1 vs. 14.8 days in Q4). *p* < 0.001. Laboratory tests showed that with the increase of BAR, the Alb level of patients continued to decrease (3.5 g/dL in Q1 vs. 2.9 g/dL in Q4, *p* < 0.001), and renal function indicators deteriorated (creatinine: 0.6 mg/dL in Q1 vs. 2.0 mg/dL in Q4; urea nitrogen: 8 mg/dL in Q1 vs. 43 mg/dL in Q4, both *p* < 0.001), hemoglobin decreased (10.6 g/dL in Q1 vs. 9.4 g/dL in Q4, *p* < 0.001), and platelet count significantly decreased in the Q4 group (211 × 10^3^/*μ*L in Q1 vs. 166 × 10^3^/*μ*L in Q4, *p* < 0.001). Comorbidities that were more frequent in the highest BAR group (Q4) were congestive heart failure (27.5% vs. 10.5% in Q1, *p* < 0.001), chronic kidney disease (35.8% vs. 8.2% in Q1, *p* = 0.001), and diabetic complications (12.7% vs. 4.8% in Q1). The final outcome showed that BAR was strongly positively correlated with mortality (31.4% in Q1 vs. 58.9% in Q4, *p* < 0.001).

**Table 1:** Baseline information for the quantile of the BAR index.

Clinic feature	Condition	BAR				*p*
		Q1 (< 3.0)	Q2 (3.0–5.0)	Q3 (5.0–9.2)	Q4 (> 9.2)	
		440	447	428	433	
Gender (%)	Female	211 (47.955)	175 (39.150)	180 (42.056)	169 (39.030)	0.024
	Male	229 (52.045)	272 (60.850)	248 (57.944)	264 (60.970)	
Los_hospital (median [IQR])		8.791 [5.459, 16.547]	10.744 [6.550, 18.889]	11.068 [6.331, 21.354]	14.799 [7.326, 26.622]	< 0.001
Albumin (median [IQR])		3.500 [3.100, 4.000]	3.100 [2.600, 3.700]	3.100[ 2.700, 3.525]	2.900 [2.400, 3.300]	< 0.001
Age (median [IQR])		53.973 [42.856, 63.617]	59.818 [49.758, 69.574]	63.635 [54.707, 74.019]	65.797 [54.172, 75.437]	< 0.001
Inr (median [IQR])		1.200 [1.100, 1.400]	1.200 [1.100, 1.400]	1.200 [1.100, 1.425]	1.300 [1.100, 1.700]	< 0.001
Pt (median[IQR])		13.200 [12.000, 15.100]	13.200 [12.150, 15.450]	13.500 [12.500, 16.025]	14.600 [12.900, 18.300]	< 0.001
Ptt (median [IQR])		29.500 [27.800, 35.600]	30.200 [27.800, 36.000]	30.100 [27.800, 37.200]	31.800 [27.800, 42.300]	0.761
Gcs (median [IQR])		15.000 [15.000, 15.000]	15.000 [15.000, 15.000]	15.000 [15.000, 15.000]	15.000 [15.000, 15.000]	< 0.001
Saps ii (median [IQR])		28.000 [21.000, 37.000]	32.000 [23.500, 43.000]	35.000 [27.000, 45.000]	44.000 [34.000, 54.000]	< 0.001
Oasis (median [IQR])		29.000 [23.000, 35.000]	31.000 [25.000, 37.000]	31.000 [26.000, 37.000]	35.000 [28.000, 42.000]	0.014
Sirs (median [IQR])		3.000 [2.000, 3.000]	3.000 [2.000, 3.000]	3.000 [2.000, 3.000]	3.000 [2.000, 3.000]	< 0.001
Anion gap (median [IQR])		12.000 [12.000, 14.000]	12.000 [11.000, 15.000]	13.000 [11.000, 15.250]	15.000 [12.000, 18.000]	< 0.001
Bicarbonate (median [IQR])		25.000 [24.000, 27.000]	25.000 [24.000, 27.000]	25.000 [22.000, 27.000]	23.000 [19.000, 25.000]	< 0.001
Bun (median [IQR])		8.000 [6.000, 9.000]	13.000 [10.000, 15.000]	20.000 [17.000, 24.000]	43.000 [33.000, 62.000]	0.016
Chloride (median [IQR])		103.000 [100.000, 104.000]	103.000 [99.000, 105.000]	103.000 [99.000, 106.000]	103.000 [98.000, 107.000]	< 0.001
Creatinine (median [IQR])		0.600 [0.500, 0.700]	0.800 [0.600, 1.000]	1.000 [0.700, 1.300]	2.000 [1.300, 3.400]	< 0.001
Glucose (median [IQR])		105.000 [100.000, 141.250]	111.000 [102.000, 141.000]	111.000 [101.000, 152.000]	126.000 [102.000, 164.000]	0.011
Sodium (median [IQR])		138.000 [136.000, 140.000]	138.000 [136.000, 140.500]	138.000 [136.000, 141.000]	138.000 [135.000, 142.000]	< 0.001
Potassium (median [IQR])		4.000 [3.600, 4.100]	4.000 [3.750, 4.300]	4.100 [3.700, 4.500]	4.200 [3.800, 4.700]	0.001
Myocardial_infarct (%)	No	419 (95.227)	406 (90.828)	379 (88.551)	384 (88.684)	
	Yes	21 (4.773)	41 (9.172)	49 (11.449)	49 (11.316)	< 0.001
Congestive_heart_failure (%)	No	394 (89.545)	382 (85.459)	340 (79.439)	314 (72.517)	
	Yes	46 (10.455)	65 (14.541)	88 (20.561)	119 (27.483)	< 0.001
Peripheral_vascular_disease (%)	No	423 (96.136)	414 (92.617)	392 (91.589)	381 (87.991)	
	Yes	17 (3.864)	33 (7.383)	36 (8.411)	52 (12.009)	0.711
Cerebrovascular_disease (%)	No	415 (94.318)	420 (93.960)	398 (92.991)	401 (92.610)	
	Yes	25 (5.682)	27 (6.040)	30 (7.009)	32 (7.390)	0.229
Chronic_pulmonary_disease (%)	No	365 (82.955)	359 (80.313)	331 (77.336)	347 (80.139)	
	Yes	75 (17.045)	88 (19.687)	97 (22.664)	86 (19.861)	0.051
Rheumatic_disease (%)	No	435 (98.864)	430 (96.197)	415 (96.963)	418 (96.536)	
	Yes	5 (1.136)	17 (3.803)	13 (3.037)	15 (3.464)	0.14
Peptic_ulcer_disease (%)	No	427 (97.045)	428 (95.749)	413 (96.495)	407 (93.995)	
	Yes	13 (2.955)	19 (4.251)	15 (3.505)	26 (6.005)	0.002
Mild_liver_disease (%)	No	312 (70.909)	344 (76.957)	339 (79.206)	302 (69.746)	
	Yes	128 (29.091)	103 (23.043)	89 (20.794)	131 (30.254)	0.048
Diabetes_without_cc (%)	No	342 (77.727)	319 (71.365)	308 (71.963)	303 (69.977)	
	Yes	98 (22.273)	128 (28.635)	120 (28.037)	130 (30.023)	< 0.001
Diabetes_with_cc (%)	No	419 (95.227)	426 (95.302)	381 (89.019)	378 (87.298)	
	Yes	21 (4.773)	21 (4.698)	47 (10.981)	55 (12.702)	0.741
Paraplegia (%)	No	435 (98.864)	439 (98.210)	420 (98.131)	428 (98.845)	
	Yes	5 (1.136)	8 (1.790)	8 (1.869)	5 (1.155)	< 0.001
Renal_disease (%)	No	404 (91.818)	385 (86.130)	327 (76.402)	278 (64.203)	
	Yes	36 (8.182)	62 (13.870)	101 (23.598)	155 (35.797)	0.001
Malignant_cancer (%)	No	378 (85.909)	366 (81.879)	376 (87.850)	393 (90.762)	
	Yes	62 (14.091)	81 (18.121)	52 (12.150)	40 (9.238)	0.004
Severe_liver_disease (%)	No	400 (90.909)	414 (92.617)	395 (92.290)	372 (85.912)	
	Yes	40 (9.091)	33 (7.383)	33 (7.710)	61 (14.088)	0.019
Metastatic_solid_tumor (%)	No	411 (93.409)	406 (90.828)	411 (96.028)	407 (93.995)	
	Yes	29 (6.591)	41 (9.172)	17 (3.972)	26 (6.005)	< 0.001
Charlson_comorbidity_index (median [IQR])		3.000 [1.000, 5.000]	4.000 [2.000, 6.000]	4.000 [3.000, 6.000]	5.000 [3.000, 7.000]	< 0.001
Hematocrit (median [IQR])		32.200 [27.600, 36.725]	31.500 [26.700, 36.500]	30.400 [26.200, 35.725]	29.200 [25.100, 34.000]	< 0.001
Hemoglobin (median [IQR])		10.600 [8.875, 12.200]	10.200 [8.600, 11.900]	9.800 [8.600, 11.700]	9.400 [8.200, 11.000]	0.018
Mch (median [IQR])		30.500 [28.800, 32.200]	30.100 [28.300, 31.600]	30.300 [28.700, 31.500]	30.500 [28.900, 31.700]	< 0.001
Mchc (median [IQR])		32.900 [32.100, 34.000]	32.700 [31.500, 33.600]	32.700 [31.500, 33.500]	32.500 [31.400, 33.500]	0.002
Mcv (median [IQR])		92.000 [87.000, 97.000]	91.000 [87.000, 95.000]	92.000 [88.000, 97.000]	92.000 [89.000, 98.000]	< 0.001
Platelet (median [IQR])		211.000 [147.750, 299.250]	222.000 [158.000, 333.500]	213.500 [150.750, 313.250]	166.000 [115.000, 262.000]	< 0.001
Rbc (median[IQR])		3.490 [3.018, 4.070]	3.490 [2.930, 4.060]	3.410 [2.888, 3.933]	3.260 [2.730, 3.670]	< 0.001
Rdw (median [IQR])		14.600 [13.700, 16.425]	15.000 [13.900, 16.700]	15.000 [14.000, 16.800]	15.600 [14.300, 17.800]	0.026
Wbc (median [IQR])		8.100 [5.800, 11.700]	8.500 [5.800, 12.850]	8.900 [5.800, 13.300]	8.900 [5.800, 14.000]	0.001
Delirium (%)	No	408 (92.727)	404 (90.380)	376 (87.850)	367 (84.758)	
	Yes	32 (7.273)	43 (9.620)	52 (12.150)	66 (15.242)	< 0.001
Outcome (%)	No	302 (68.636)	290 (64.877)	247 (57.710)	178 (41.109)	
	Yes	138 (31.364)	157 (35.123)	181 (42.290)	255 (58.891)	< 0.001
BAR (median [IQR])		2.273 [1.775, 2.727]	4.000 [3.478, 4.577]	6.593 [5.758, 7.731]	14.400 [11.389, 22.143]	< 0.001

*Note:* Continuous variables are expressed as mean ± standard deviation, and categorical variables are expressed as number (%).

K–M survival analysis (Figure 2) showed that there were significant differences in all‐cause mortality among different BAR groups (log‐rank *p* = 0.035). Among them, survival outcomes of group Q4 (BAR ≥ 9.2, *n* = 433) was the worst, and the survival rate dropped to 43% at 40 days, which was significantly lower than that of group Q1 (BAR < 3.0, 78% of *n* = 440) (hazard ratio HR = 1.82); the survival curves of each group showed a clear stratified trend (Q1 > Q2 > Q3 > Q4), and the early survival rate decreased sharply (0–20 day mortality rate: 63.5% in Q4 group vs. 18.0% in Q1 group). After 60 days, the decline slowed down, but the differences between groups persisted (60‐day survival rate: 92% in Q1 group vs. 83% in Q4 group). Patients in the high BAR group not only had the highest initial risk of death (433 cases), but also had the highest event density (with a decline of 96.1% within 60 days), indicating a dose‐dependent positive correlation between BAR levels and the risk of all‐cause mortality.

**Figure 2:**
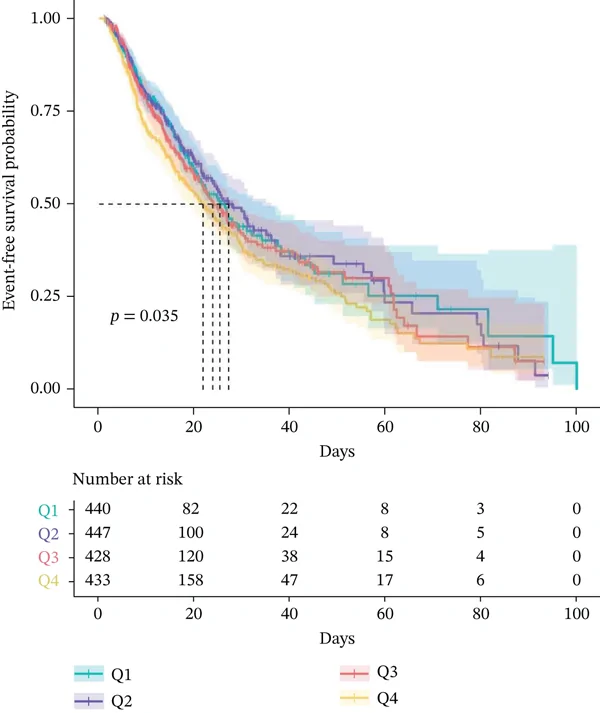
Kaplan–Meier survival curves for all‐cause mortality across BAR quantile groups (log‐rank *p* = 0.035).

In this study, the analysis results based on the multiple regression model show that as the BAR level increases, the risk of delirium and all‐cause mortality in patients significantly increases in unadjusted models (Table 2). Binary logistic regression analysis of the outcome of delirium revealed that when BAR was used as a continuous variable, even after fully adjusting for demographic characteristics (age, gender), acute disease severity scores (GCS, SAPS II, OASIS, SIRS), Charlson comorbidity index, and 17 specific comorbidities (including cardiovascular and cerebrovascular diseases, diabetes, liver, and kidney dysfunction), for every 1‐unit increase in BAR, the risk of delirium still significantly increased by 2.7% (adjusted odds ratio (OR) = 1.027, 95% confidence interval (CI): 1.009–1.044, *p* = 0.002); When BAR was grouped by quartiles, the risk in the highest quartile group (Q4) was also significantly higher than that in the lowest group (Q1) (adjusted OR = 1.882, 95% CI: 1.157–3.108, *p* = 0.012). However, when the Cox proportional hazards model was used to analyze all‐cause mortality, the association between BAR and mortality risk presented different patterns: although in the unadjusted model, the increase in BAR per unit and the highest quartile group showed a weak but significant increase in risk (e.g., HR = 1.278 in group Q4, *p* = 0.022), this association completely disappeared after stepwise correction (especially after adjusting for acute disease score and overall comorbidity burden) (HR = 1.022 in group Q4 in the final adjusted model, *p* = 0.800), indicating that its initial association was mainly driven by confounding factors. In conclusion, BAR is an independent risk factor for delirium, but it has no significant independent association with all‐cause mortality after multivariable adjustment.

**Table 2:** Cox regression and logistic Regression for all‐cause mortality and delirium.

	Model 1		Model 2		Model 3		Model4	
	Logistic Regression							
Categories	OR (95% CI)	*p*‐value	OR(95% CI)	*p*‐value	OR (95% CI)	*p*‐value	OR (95% CI)	*p*‐value
Delirium								
Continues variable per unit	1.032 (1.017, 1.048)	< 0.001	1.038 (1.022, 1.054)	< 0.001	1.028 (1.010, 1.044)	0.001	1.027 (1.009, 1.044)	0.002
Quartile								
Q1	**——**	**——**	**——**	**——**	**——**	**——**	**——**	**——**
Q2	1.357 (0.844, 2.203)	0.2	1.534 (0.948, 2.506)	0.083	1.332 (0.813, 2.200)	0.3	1.327 (0.808, 2.197)	0.3
Q3	1.763 (1.117, 2.823)	0.016	2.086 (1.303, 3.387)	0.002	1.853 (1.148, 3.032)	0.012	1.817 (1.120, 2.984)	0.017
Q4	2.293 (1.481, 3.617)	< 0.001	2.812 (1.781, 4.521)	< 0.001	1.932 (1.197, 3.169)	0.008	1.882 (1.157, 3.108)	0.012
	Cox regression							
All‐cause mortality								
Categories	HR (95% CI)	*p*‐value	HR (95% CI)	*p*‐value	HR (95% CI)	*p*‐value	HR (95% CI)	*p*‐value
Continues variable per unit	1.013 (1.006, 1.020)	< 0.001	1.007 (1.000, 1.015)	0.046	1.006 (0.999, 1.014)	0.01	1.008 (1.000, 1.015)	0.043
Quartile								
Q1	——	——	——	——	——	——	——	——
Q2	0.989 (0.786, 1.244)	> 0.9	0.860 (0.682, 1.085)	0.2	0.896 (0.709, 1.132)	0.4	0.907 (0.717, 1.149)	0.4
Q3	1.093 (0.875, 1.366)	0.4	0.855 (0.680, 1.074)	0.2	0.931 (0.741, 1.171)	0.5	0.953 (0.756, 1.203)	0.7
Q4	1.278 (1.037, 1.575)	0.022	0.999 (0.806, 1.239)	> 0.9	0.988 (0.795, 1.228)	> 0.9	1.022 (0.817, 1.279)	0.8

*Note:* MODEL 1: none; MODEL 2: age and gender: MODEL 3: age, gender, GCS, SAPSII, OASIS, SIRS, CHARLSON score; MODEL 4: age, gender, GCS, SAPSII, OASIS, SIRS, CHARLSON score, myocardial infarct, congestive heart failure, peripheral vascular disease, cerebrovascular disease, dementia, chronic pulmonary disease, rheumatic disease, peptic ulcer disease, mild liver disease, diabetes without chronic comorbidities, diabetes with chronic comorbidities, paraplegia, renal disease, malignant cancer, severe liver disease, and metastatic solid tumor.

RCS analysis showed a nonlinear association between BAR levels and clinical outcomes (Figure 3). In the logistic regression model of delirium outcomes (Figure 3A–D), when BAR < 4.0, every 1‐unit decrease in BAR was associated with an increased risk of delirium (OR = 1.15 when BAR = 2.0, 95% CI: 1.05–1.26), and the risk remained stable when BAR = 4.0–6.0 (OR≈1). When BAR > 9.0, the risk increases sharply (OR = 2.05 when BAR = 12.0, 95% CI: 1.78–2.36). The Cox regression model for all‐cause mortality outcomes (Figure 3E–H) presented different patterns. The risk decreased significantly when BAR < 3.5 (HR = 0.85 at BAR = 2.0, 95% CI: 0.76–0.94), and continued to increase linearly after BAR > 3.5 (HR = 1.92 at BAR = 12.0). 95% CI: 1.65–2.24, but in the high‐value area of BAR > 9.0, it shows a platformization trend (the fluctuation increases after the upper limit of the 95% CI exceeds 2.0). The two models jointly indicate that there is a threshold effect in the nonlinear relationship between BAR and the outcome: The risk of delirium was U‐shaped associated in the low BAR area (< 4.0), whereas the risk of death showed a continuously increasing dose–response relationship after > 3.5 (HR increased by 1.12–1.15 for every 1‐unit increase), and BAR > 9.0 was determined as the clinical high‐risk critical value (delirium OR > 1.78, HR of death > 1.97).

**Figure 3:**
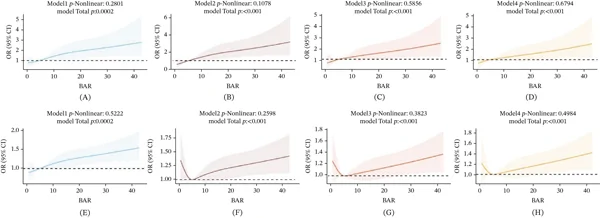
Restricted cubic spline curves for the associations of BAR with all‐cause mortality and delirium. (A–D) Associations between BAR and delirium risk in four logistic regression models. (E–H) Associations between BAR and all‐cause mortality in four Cox regression models.

In logistic regression subgroup analyses (Figure 4A) for delirium, higher BAR levels were significantly associated with increased delirium risk overall (OR 1.03, 95% CI 1.01–1.04, *p* < 0.001), with consistent positive associations observed across most subgroups including both age strata and genders; significant effect modification emerged for diabetes with chronic comorbidities (*p* − interaction = 0.023) and renal disease (*p* − interaction = 0.002), where the BAR‐associated risk was attenuated in patients with these conditions. In Cox regression subgroup analyses for all‐cause mortality (Figure 4B), BAR demonstrated a weaker but statistically significant overall association with mortality risk (HR 1.01, 95% CI 1.00–1.01, *p* = 0.015), with significant risk increases primarily observed in older patients (> 65 years), females, and those without diabetes with chronic comorbidities; a trend toward effect modification by age was noted (*p* − interaction = 0.059), with BAR‐associated mortality risk confined to the older subgroup. The magnitude of BAR′s effect was substantially greater for delirium than for mortality across most comparative subgroups.

**Figure 4:**
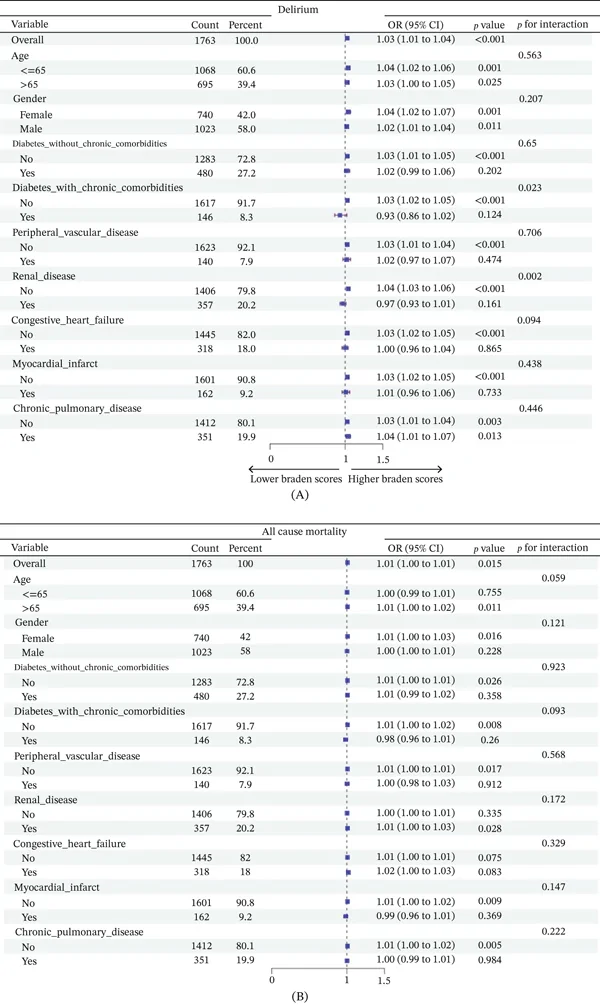
Forest plots of subgroup analyses for delirium and all‐cause mortality. Subgroups included age, gender, diabetes, peripheral vascular disease, renal disease, congestive heart failure, myocardial infarction, and chronic pulmonary disease. (A) Outcome: delirium. (B) Outcome: all‐cause mortality.

In all‐cause mortality, the ROC curve compares two models (Figure 5A). The red line (numeric BAR) has an AUC of 0.615 (95% CI: 0.557–0.670), whereas the blue line (BAR‐IQR; patients stratified by BAR quartiles; IQR) has a slightly higher AUC of 0.635 (95% CI: 0.573–0.693). Both models demonstrate modest discriminatory ability, with the BAR‐IQR‐based model performing slightly better. In delirium, the ROC curve compares two models (Figure 5B). The red line (numeric BAR) has an AUC of 0.596 (95% CI: 0.488–0.696), whereas the blue line (BAR‐IQR) has an AUC of 0.599 (95% CI: 0.483–0.716). Both models show moderate performance, with the BAR‐IQR‐based model slightly outperforming the numeric one. The AUC values indicate that BAR has modest predictive ability for these outcomes.

**Figure 5:**
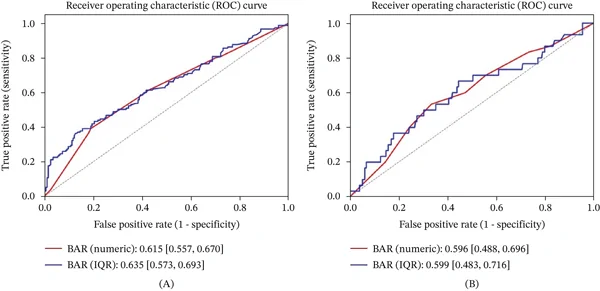
ROC curves for predictive performance of BAR. (A) Continuous and categorized BAR for predicting all‐cause mortality. (B) Continuous and categorized BAR for predicting delirium.

## 4. Discussion

In this study, we systematically evaluated the predictive value of the BAR for mortality and delirium risk in pancreatitis patients using retrospective data from the MIMIC‐IV database. Our findings demonstrated that both all‐cause mortality and delirium incidence increased significantly with rising BAR levels in unadjusted analyses. Patients in the highest BAR quartile exhibited substantially higher mortality and delirium rates compared to those in the lowest quartile. Correspondingly, disease severity scores worsened significantly with elevated BAR. Kaplan–Meier analysis further revealed that survival probability declined most rapidly in the high BAR group, with complete mortality observed. These findings suggest BAR′s potential as a comprehensive marker of inflammatory and metabolic status in pancreatitis patients for prognostication and its value for unadjusted prognostication, particularly for delirium.

Previous studies have frequently examined BUN and Alb independently as predictors of all‐cause mortality. BUN is one of the most established biomarkers in AP. Early evidence from a landmark multicenter study demonstrated that BUN elevation within 24 h of admission is independently associated with increased mortality, whereas subsequent BUN decline correlates with improved survival [22]. Subsequent studies have consistently confirmed this association. Li et al. reported a 5% increase in 30‐day all‐cause mortality risk for every 10 mg/dL rise in BUN after full adjustment (HR = 1.05; 95% CI: 1.02–1.07; *p* < 0.001) [23]. In all‐cause mortality of pancreatitis, the level of serum BUN elevated could predict mortality and persisting multiorgan failure [24]. More recently, Kurtipek et al. identified BUN as the most prominent marker for early prediction of severe AP, further reinforcing its clinical utility as a simple and practical biomarker [25].

Alb, synthesized by the liver, tends to decrease more markedly than serum creatinine in critically ill patients, making it a valuable prognostic indicator in pancreatitis [26]. Similarly, a study in a Chinese cohort demonstrated that persistently low serum Alb was associated with poorer pancreatitis outcome [27]. Hypoalbuminemia in pancreatitis is primarily caused by endotoxin‐mediated inhibition of Alb synthesis and increased capillary leakage [28, 29]. However, after adjustment for confounders, the association with mortality was not independent, suggesting that Alb reflects overall illness severity rather than providing unique predictive information for mortality. Stratified analyses and adjustments for potential confounders validated BAR as a robust prognostic marker for pancreatitis mortality risk. As reported by recent multiple studies published in 2025, have consistently shown that elevated BAR is independently linked to increased 28‐day, 90‐day, 1‐year, and inhospital mortality in AP patients, with predictive efficacy comparable to or better than SOFA, APACHE II, and SAPS II scores [30–34]. Additionally, Kuang et al. further confirmed that BAR could greatly improve the prognostic predictive accuracy of single BUN and ALB indicators, presenting excellent predictive value, particularly in patients with biliary AP [35]. In coronavirus disease 2019, researchers identified BAR as a robust predictor of mortality compared with BUN and Alb, reporting an area under the receiver operating characteristic (ROC) curve of 0.809 [36]. Likewise, a multicenter Korean study found BAR to be a novel predictor of bacteremic febrile urinary tract infections in young infants, with an area under the ROC curve of 0.759 [37]. Collectively, these findings underscore the reliability and cross‐population robustness of BAR as a prognostic indicator in conditions characterized by systemic inflammation. Although these studies were primarily conducted in infectious diseases, BAR elevation in those settings is largely driven by inflammatory responses—a pathway that is similarly activated in AP. Our findings extend this to delirium prediction in pancreatitis. Compared with traditional multifactorial scoring systems such as Ranson′s criteria, BISAP, and APACHE‐II, BAR offers several distinct advantages. It is simpler, more cost‐effective, and more readily available in emergency settings, requiring only two routinely measured laboratory parameters, making it particularly suitable for rapid risk stratification in resource‐limited clinical environments. These features support the potential clinical utility of BAR as a practical and accessible tool for early identification of high‐risk pancreatitis patients.

Another important finding of this study is that BAR is an effective and independent predictor of delirium risk in patients with pancreatitis. Delirium is a common symptom of a significant contributor to its poor prognosis. However, until now, the relationship between BAR and delirium risk in pancreatitis has remained unexplored, with prior research generally focusing on single biomarkers or other disease contexts. For example, a small single‐center study in Turkey identified elevated urea levels as an independent biomarker of delirium in patients with COVID‐19, reporting an adjusted OR of 0.015 (95% CI: 0.058–0.032) [38]. Fang et al. demonstrated a without renal failure, with patients having BUN levels exceeding 23 mg/dL showing a 35%–39% increased risk of delirium (OR = 1.35–1.39, all *p* < 0.001) [39]. Similarly, Newman et al. showed that urea nitrogen was the only laboratory marker with predictive value for delirium in their risk‐stratification model [40]. Proposed mechanisms underlying urea nitrogen′s association with delirium include its neurotoxicity, cerebrovascular endothelial damage, inhibition of microvascular endothelial proliferation, and induction of insulin resistance [41–45]. The prognostic value of Alb for delirium remains controversial. Wang et al.′s study in a Chinese Han population identified low serum Alb as a risk factor for delirium following knee or hip arthroplasty [46], whereas Chi et al. found no significant correlation between Alb levels and delirium incidence, duration, or severity in critically ill adults in the ICU [47].

Wang et al. examined the association between BAR and mortality in patients with renal disease. The HRs for 30‐day all‐cause mortality decreased from 1.97 (95% CI: 1.76–2.20) to 1.32 (95% CI: 1.04–1.67) after adjustment, whereas the HRs for 360‐day mortality decreased from 1.78 (95% CI: 1.63–1.96) to 1.55 (95% CI: 1.28–1.88); the interaction p‐values were 0.267 and 0.019, respectively [20]. A recent multicenter prospective study demonstrated that mortality risk prediction using machine‐learning algorithms significantly reduced inhospital mortality, length of hospital stay, and 30‐day readmission rates. Notably, the reduction in hospital stay alone resulted in an average cost savings of $42,300 per patient [48]. This underscores the critical importance of identifying key risk indicators for pancreatitis mortality prediction, a need directly addressed by our study. BUN and Alb are routinely measured, widely available, and cost‐effective clinical parameters. Our analysis based on the MIMIC‐IV database confirms the validity and reliability of their BAR as a predictor of pancreatitis mortality and delirium risk. Consequently, BAR holds promise as a novel prognostic biomarker and may serve as a valuable addition to existing pancreatitis risk stratification tools, including future clinical scoring systems and machine‐learning models. However, before BAR can be integrated into routine clinical practice, several tasks remain essential. These include comparative evaluations of BAR alongside traditional markers such as lactate, as well as recently proposed indicators like the BUN to serum Alb ratio and fever, within the same patient cohorts. Additionally, external validation across multiple centers and assessment of the associated prognostic and economic benefits are necessary.

The primary strengths of our study include the large patient cohort and the novel application of BAR for predicting pancreatitis delirium risk. The primary strengths of our study include the large patient cohort derived from a public database and the novel application of BAR for predicting delirium risk specifically in pancreatitis. Nonetheless, several important limitations must be acknowledged. First, the retrospective, single‐center design may limit the generalizability of our findings. Second, several patients with chronic and recurrent pancreatitis were enrolled in this study. Although this design broadens the application scope of our findings, it also reduces the disease specificity and clinical pertinence of the results to a certain extent. Third, we did not conduct an in‐depth stratified discussion based on different etiological types of pancreatitis, such as gallstone and alcoholic pancreatitis. Further stratified research is needed to more clearly explore the influence of different causes on clinical outcomes, including delirium. Fourth, the timing of BUN and Alb measurements relative to disease onset or ICU admission was not standardized, which may affect the interpretation of dynamic changes and their prognostic value. Fifth, the predictive performance of BAR, as reflected by the AUC values, was moderate. Although relevant studies have compared BAR with well‐established prognostic scores, including APACHE II, SOFA, and BISAP, further head‐to‐head investigations would help better clarify its incremental clinical utility. Tie et al. reported that hypoproteinemia at ICU admission does not directly predict outcomes for all patients; instead, longitudinal changes in Alb during treatment warrant greater attention [49]. Although we adjusted for numerous confounders, residual confounding cannot be excluded.

In summary, this study confirms that BAR can independently predict delirium risk and reflect disease severity among patients with pancreatitis. Consistent with part of previous findings, BAR failed to show a significant independent correlation with mortality after multivariate adjustment. As a convenient and easily accessible indicator, BAR is suitable for auxiliary risk stratification, especially in screening patients at high risk of delirium. Further multicenter prospective studies, head‐to‐head comparison with classic prognostic scoring systems, and external validation are still required to fully verify its clinical application value and explore its advantages in prognostic evaluation.

**Nomenclature**
Alb: albumin
AP: acute pancreatitis
APACHE II: Acute Physiology and Chronic Health Evaluation II
BISAP: Bedside Index of Severity in Acute Pancreatitis
BAR: Blood Urea Nitrogen‐to‐Albumin Ratio
BUN: Blood Urea Nitrogen
CI: confidence interval
CITI: Collaborative Institutional Training Initiative
EPC: extrapancreatic complication
GBD: Global Burden of Disease
GCS: Glasgow Coma Scale
HR: hazard ratio
ICD: International Classification of Diseases
ICU: intensive care unit
INR: International Normalized Ratio
IQR: interquartile range
K‐M: Kaplan–Meier
LOS: length of stay
MIMIC‐IV: Medical Information Mart for Intensive Care‐IV
MCH: Mean Corpuscular Hemoglobin
MCHC: Mean Corpuscular Hemoglobin Concentration
MCV: Mean Corpuscular Volume
OASIS: Oxford Acute Severity of Illness Score
OR: odds ratio
PT: prothrombin time
PTT: partial thromboplastin time
RBC: red blood cell
RDW: red cell distribution width
RCS: restricted cubic spline
SOFA: Sequential Organ Failure Assessment
SAPS II: Simplified Acute Physiology Score II
SIRS: systemic inflammatory response syndrome
VIF: variance inflation factor
WBC: white blood cell count

## Author Contributions

S.D. and J.X. wrote the main manuscript text and revised the manuscript. Y.Z. assisted with data collection and statistical analysis. P.Y. and Q.W. supervised the research and revised the manuscript critically.

## Funding

No funding was received for this manuscript.

## Disclosure

All authors read and approved the final manuscript.

## Ethics Statement

This research used the publicly available MIMIC‐IV (v. 3.0) database, which contains deidentified data. Secondary analysis of these anonymized records proceeded under applicable regulatory policies, negating the necessity for new institutional ethics board approval or individual consent acquisition.

## Consent

The authors have nothing to report.

## Conflicts of Interest

The authors declare no conflicts of interest.

## Data Availability

No datasets were generated or analyzed during the current study.
